# Supplementary material for: High-contrast, speckle-free, true 3D holography via binary CGH optimization
Source: Sci Rep. 2022 Feb 18;12:2811. doi: 10.1038/s41598-022-06405-2 (PMC8857227; doi:10.1038/s41598-022-06405-2)
Supplement: Supplementary file 1 — Supplementary Information. [file 41598_2022_6405_MOESM1_ESM.pdf]

Supplementary information

High-contrast, speckle-free, true 3D holography via binary CGH optimization

Byounghyo Lee, Dongyeon Kim, Seungjae Lee, Chun Chen, and Byoungho Lee*

School of Electrical and Computer Engineering, Seoul National University, Gwanak-Gu Gwanakro 1, Seoul 08826, South Korea

*byoungho@snu.ac.kr

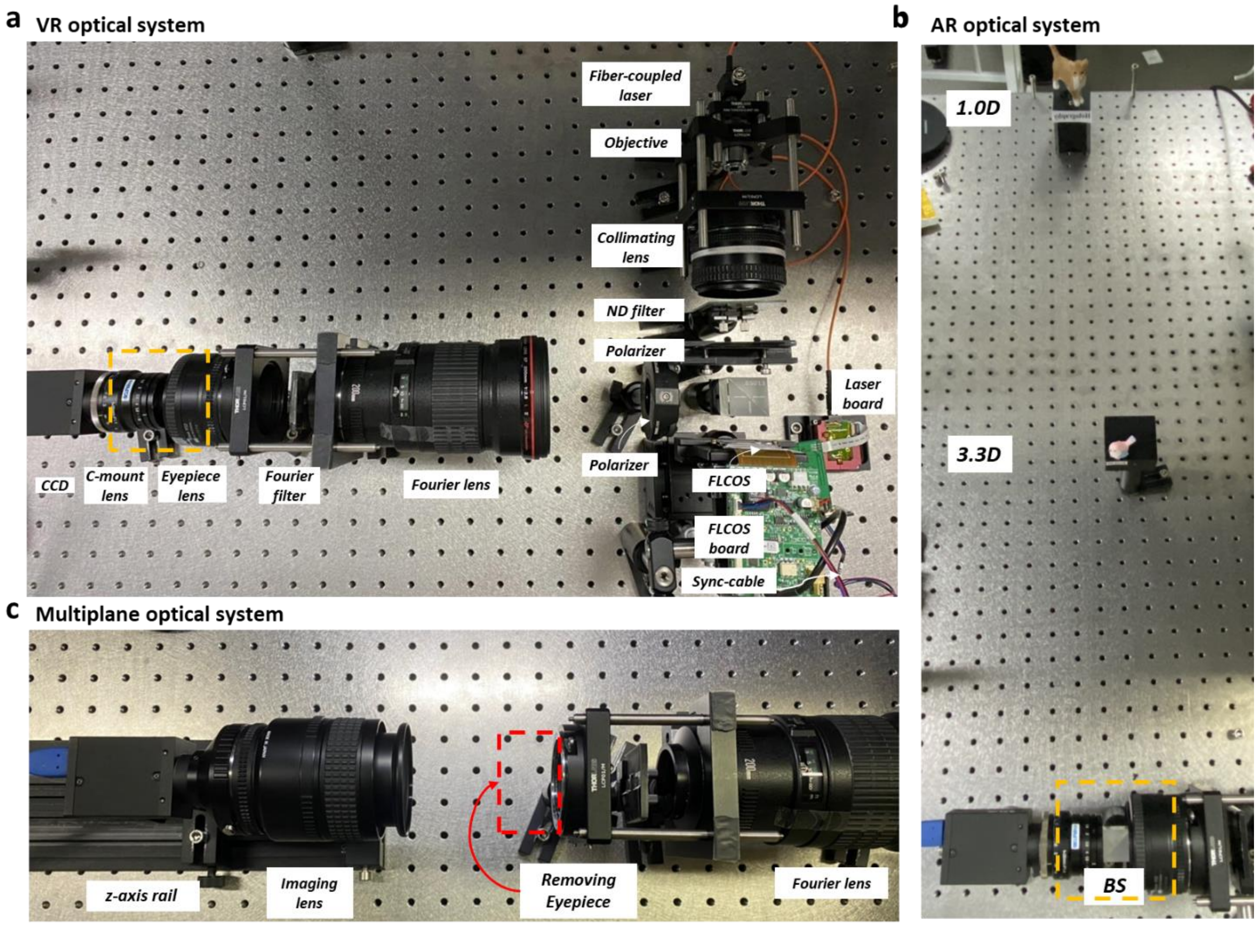

Supplementary Figure 1. Photograph of the true 3D holographic prototypes. a. VR optical system for near-eye displays. **b.** AR optical system for near-eye displays implemented with an additional BS. **c.** Muitiplane projection system, constructed by removing the eyepiece lens of (a). The multiplane intensities are captured by axially moving the camera using the *z*-axis rail.

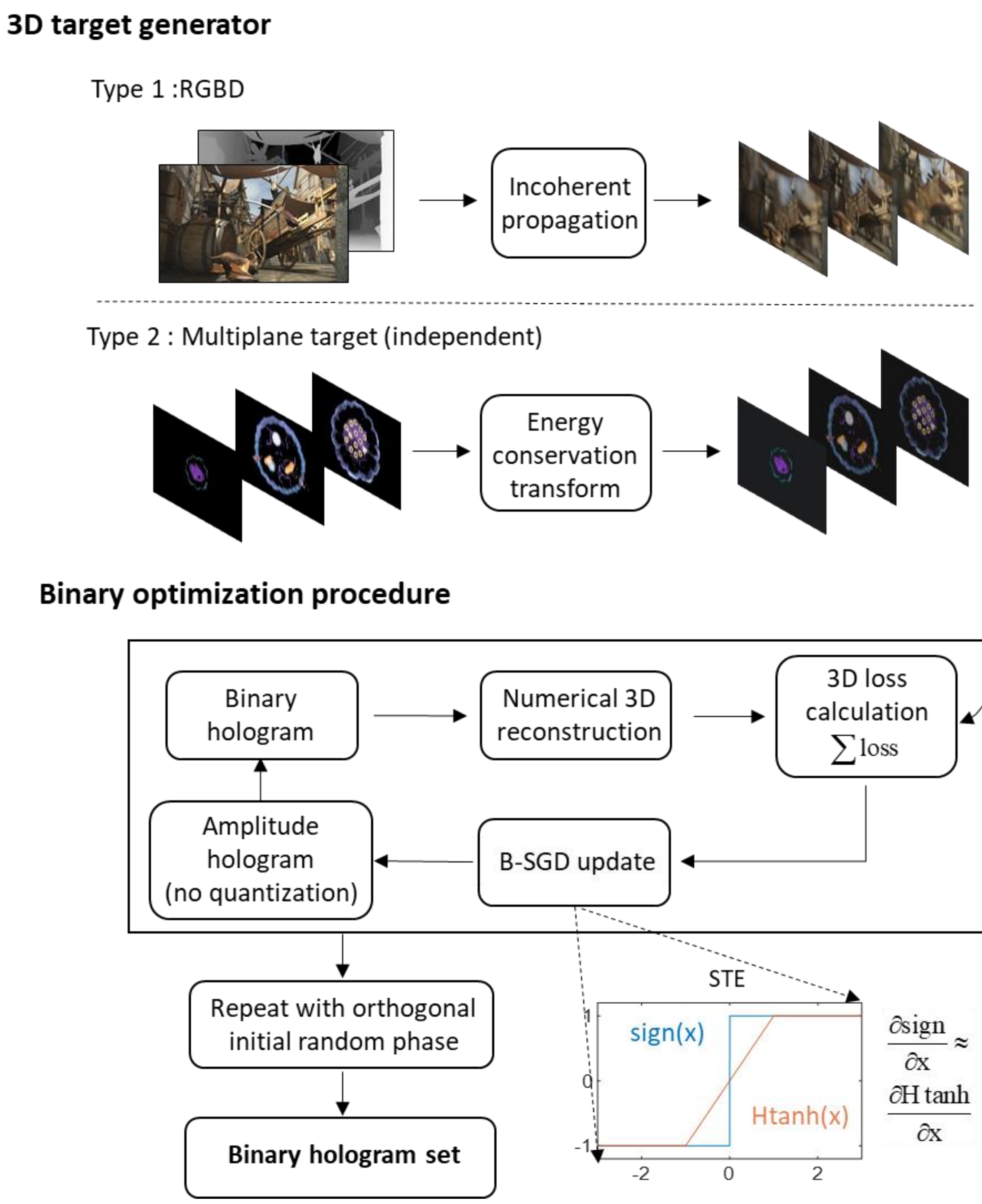

Supplementary Figure 2. Binary optimization framework. Procedure of generating true 3D CGH. Accurate 3D target scenes are calculated according to the types of inputs that RGBD or multiplane images. We develop the robust binary CGH optimization method, optimizing a binary hologram to minimize amplitude differences for all depth layers. The source images by © copyright Blender Foundation | durian.blender.org

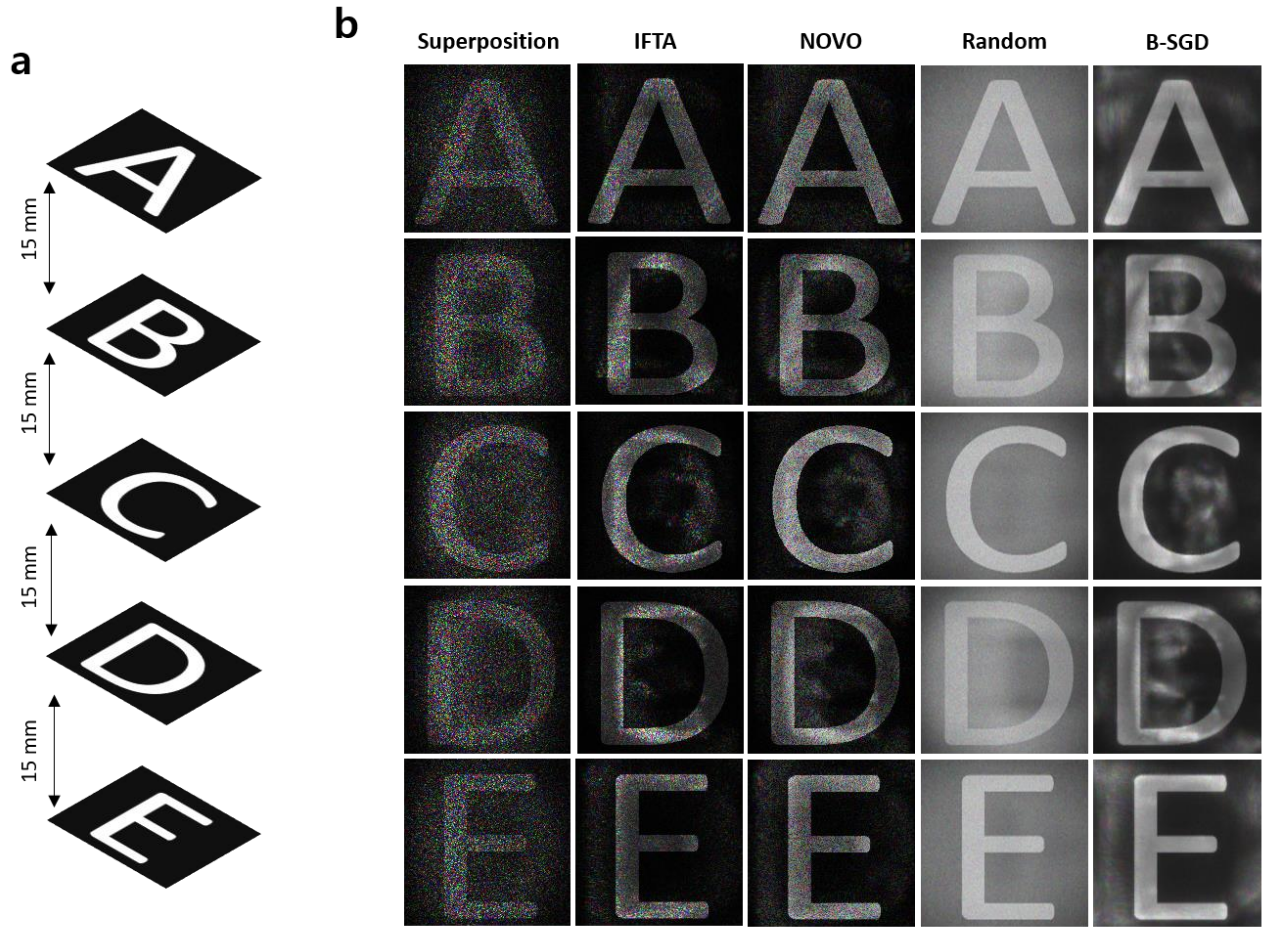

Supplementary Figure 3. Performance comparison for crosstalk-free 3D projection using characters. a. Target 3D intensity distribution that five characters spaced 15 mm apart. **b.** Simulated reconstructed intensity at each target depth. Compared to superposition, IFTA[4] and NOVO[5] sequentially improve performance with less crosstalk noise. Random (simply multiplexed binary holograms) gives speckle free results, but there is a lot of back ground noise from crosstalk and binary noise. B-SGD provides best quality results with no speckle and less crosstalk.

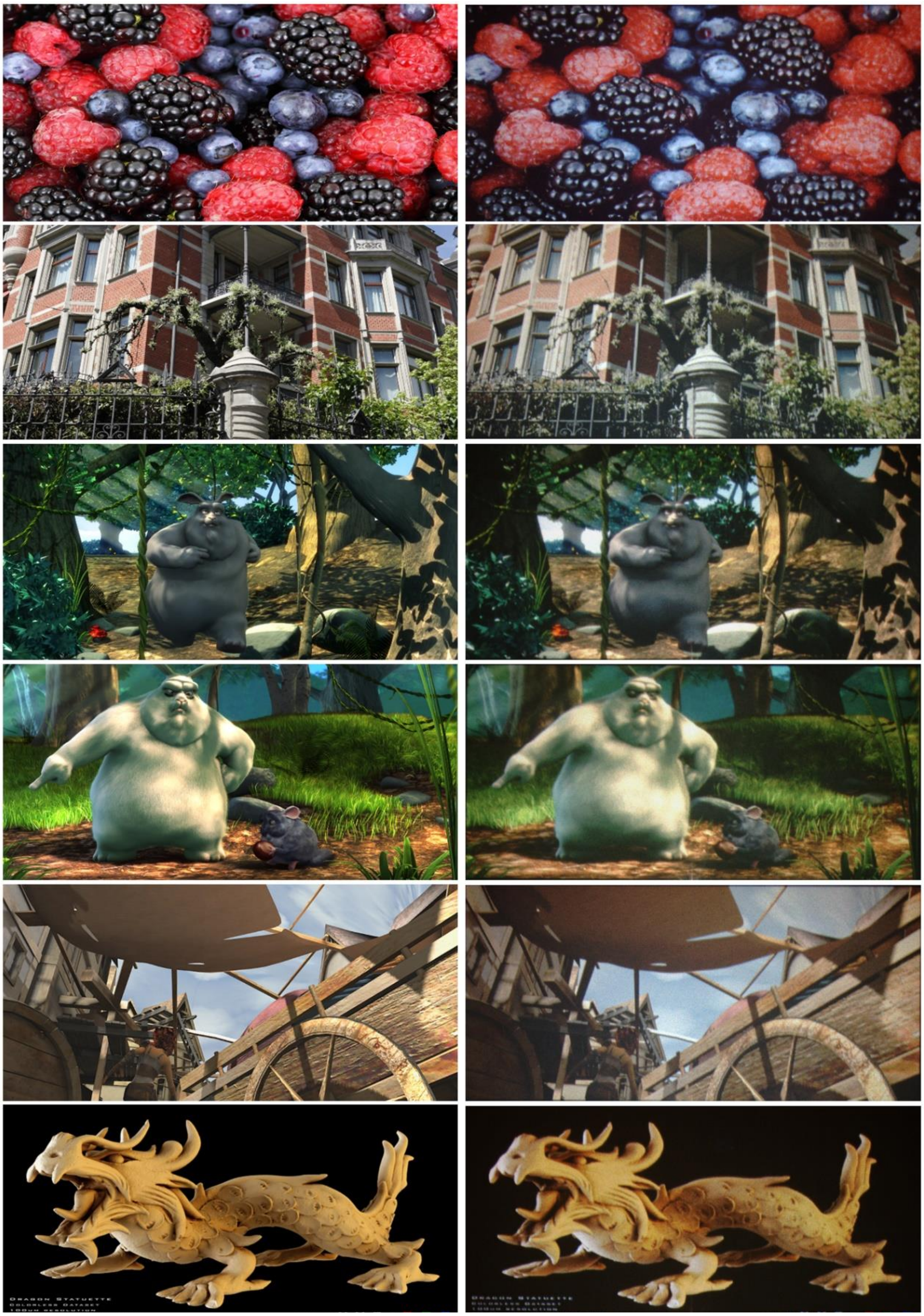

Supplementary Figure 4. Experimental results of true 3D holography in 2D case. (left) Target images. (right) Experimental results. The source images by Shutterstock[1], All Right Reserved, Kim. C. et al.[2], Stanford 3D scanning repositor[3], © copyright 2008 Blender Foundation | www. bigbuckbunny.org, and © copyright Blender Foundation | durian.blender.org.

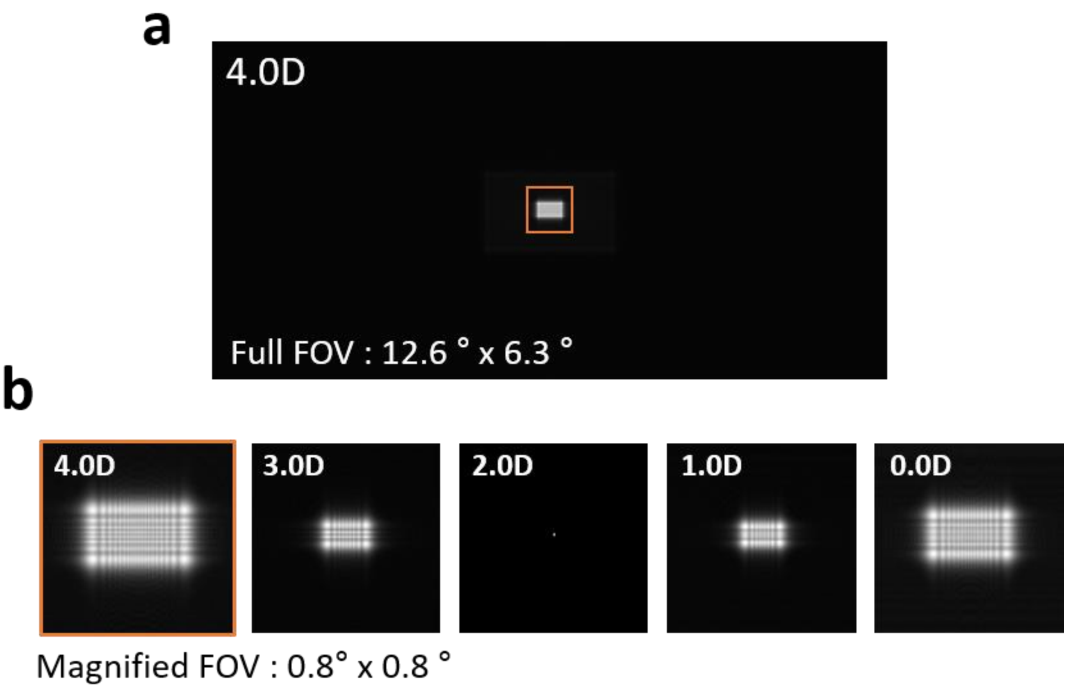

Supplementary Figure 5. Simulated point spread function (psf) of the near-eye display system. The target depth of the point source is 2.0D. **a.** Full field of view (FOV) image at the 4.0D. **b.** Magnified images at the different propagation distances (4.0D to 0.0D). The shape of psf follows a rectangular form due to the shape of the eyebox, which is determined by the physical structure of the SLM.

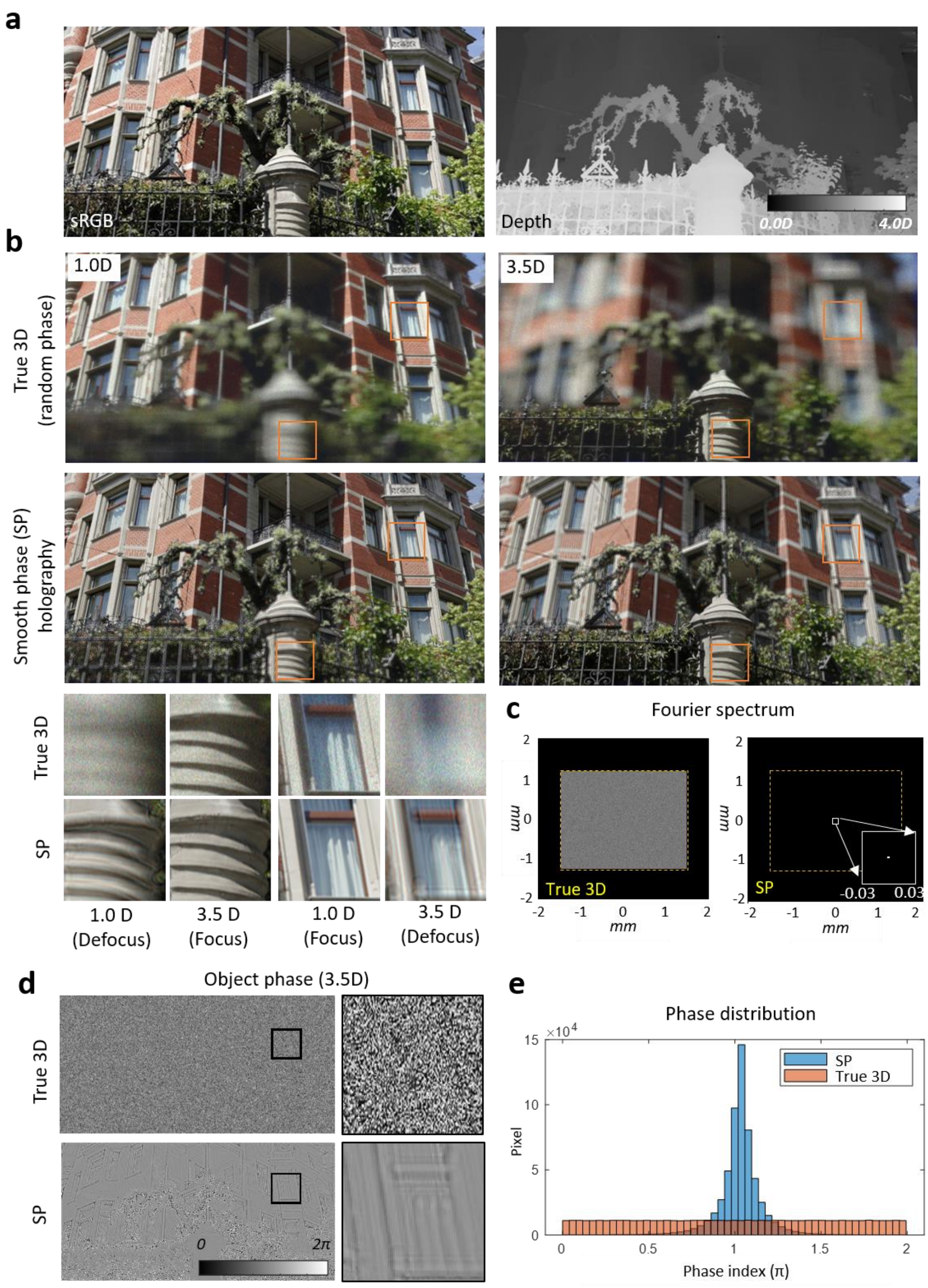

Supplementary Figure 6. Analysis of the object phase distribution for 3-D holography. a. Target RGBD **b.** Simulation results of our method and the SP holography. Our random phase based holography provides realistic focus cues. SP holography has low-axial resolution, so its 3D scenes are almost all-in-focus. **c.** Fourier spectrum of both phase distributions where the orange dot lines correspond to the maximum diffraction angle of the SLM. **d.** Reconstructed object phases of the 3.5D focused scenes. Red channel phases are represented in the gray scale. **e.** Histogram of the reconstructed object phase distributions presented in part (d). The 3D data by Kim, C. et al.[2].

Description of Supplementary Videos

Supplementary Video 1: The video shows axial sweep results of multiplane projection system. The test images by Kim, C. et al.[2], Sheikh, H.R. et al.[6], and © copyright 2008, Blender Foundation | www.bigbuckbunny.org.

Supplementary Video 2: The video shows focal sweep of 3D scenes by using the near-eye display system. The 3D scenes by © copyright Blender Foundation | durian.blender.org.

Supplementary Video 3: The video shows 50 Hz focal sweep results of 3D scene. The video also shows 20 Hz replay video of the 50 Hz recorded results as focusing on the near, middle, and far depths. The 3D scenes by © copyright Blender Foundation | durian.blender.org.

Supplementary Video 4: The video shows AR focal sweep results, recorded with real-objects.

Supplementary Video 5: The video shows AR parallax sweep results, recorded with real-objects.

References

1. https://www.shutterstock.com/

2. Kim, C., Zimmer, H., Pritch, Y., Sorkine-Hornung, A. & Gross, M. H. Scene reconstruction from high spatio-angular resolution light fields. *ACM Trans. Graph*. **32**, 73-1 (2013).

3. http://graphics.stanford.edu/data/3Dscanrep/

4. Makey, G.et al. Breaking crosstalk limits to dynamic holography using orthogonality of high-dimensional random vectors. *Nat. Photonics* **13**, 251–256 (2019).

5. Zhang, J., Pégard, N., Zhong, J., Adesnik, H. & Waller, L. 3d computer-generated holography by non-convex optimization. *Optica* **4**, 1306–1313 (2017).

6. Sheikh, H. R., Sabir, M. F. & Bovik. A. C. A statistical evaluation of recent full reference image quality assessment algorithms. *IEEE Trans. on image processing* **15**, 3440-3451 (2006).
